# Long-Term Neurodevelopmental Outcome of Children after in Utero Exposure to Chemotherapy

**DOI:** 10.3390/cancers12123623

**Published:** 2020-12-03

**Authors:** Anna-Maria Korakiti, Eleni Zografos, Mathilde van Gerwen, Frédéric Amant, Meletios-Athanasios Dimopoulos, Flora Zagouri

**Affiliations:** 1 Department of Clinical Therapeutics, Alexandra Hospital, School of Medicine, National and Kapodistrian University of Athens, 11528 Athens, Greece; annamaria_kor@hotmail.com (A.-M.K.); el_zogra@hotmail.com (E.Z.); mdimop@med.uoa.gr (M.-A.D.); 2 Center for Gynecologic Oncology Amsterdam, Antoni van Leeuwenhoek–Netherlands Cancer Institute, 1066 CX Amsterdam, The Netherlands; m.v.gerwen@nki.nl (M.v.G.); f.amant@nki.nl (F.A.); 3 Princess Máxima Center for Pediatric Oncology, 3584 CS Utrecht, The Netherlands; 4 Department of Oncology, KU Leuven, 3000 Leuven, Belgium; 5 Center for Gynecologic Oncology Amsterdam, Amsterdam UMC, University of Amsterdam, 1105 AZ Amsterdam, The Netherlands

**Keywords:** neurodevelopment, child development, pregnancy, maternal cancer, chemotherapy

## Abstract

**Simple Summary:**

Facing cancer diagnosis during pregnancy constitutes a truly complex and challenging situation for both the patients and the physicians. Cancer diagnosis in a period of hope and joy is an unendurable situation that may affect the psychosocial functioning of the mother, causing depression, anxiety, self-blame, and social isolation. At the same time, a moral dilemma evolves among medical professionals; what is best for the mother in terms of immediate chemotherapy may have detrimental effects on the fetus, and conversely, delaying therapy and protecting the fetus may have a negative impact on the mother as the tumor progresses. Solid data on the safety profile or risks of anti-cancer agents and on the long-term neurodevelopmental outcome of children after in utero exposure to chemotherapy may provide both the patients and the physicians the information necessary for shared decision making when cancer is diagnosed during pregnancy.

**Abstract:**

Pregnancy-related cancer management represents a real challenge for both the patients and the physicians. The long-term neurodevelopmental outcome of children in utero exposed to chemotherapeutic agents has only recently been addressed. This review aims to systematically integrate and highlight all existing data from the literature regarding the effect of prenatal exposure to chemotherapy on fetal brain growth and child development. All eligible studies are based on validated neurodevelopmental testing scales (e.g., Bayley Scales of Infant Development, Wechsler Preschool and Primary Scale of Intelligence) and/or well-defined questionnaires. Our systematic review including 17 studies demonstrates that no major consequences on the neurodevelopment of children after in utero exposure to anti-cancer drugs have been reported; nevertheless, longer and more thorough follow-up with large-scale multicenter prospective studies is certainly required in order to draw firm conclusions.

## 1. Introduction

Facing cancer during pregnancy imposes a complex and challenging situation for patients and physicians. On the one hand, a life-threatening disease and the uncertainty about pregnancy may trigger symptoms of psychological distress in the mother to be. On the other hand, an ethical dilemma evolves among medical professionals; toxic and immediate chemotherapy may have detrimental effects on the developing fetus, whereas delaying cancer treatment may permit further tumor progression and result in inferior oncologic outcome.

Pregnancy-related cancer affects approximately 1 in 1000 women every year [1,2]. Breast cancer, cervical cancer, hematological cancer, and melanoma represent the most frequently diagnosed malignancies during gestation [1,2,3]. As women tend to defer childbearing to a later age, pregnancy-associated cancer incidence is expected to increase severely in developing countries in the upcoming years [4,5]. Furthermore, the introduction of non-invasive prenatal testing (NIPT) aiming to identify fetal chromosomal abnormalities in obstetrical care has resulted in a further increase in cancer detection in asymptomatic pregnant patients in the developed countries [6,7].

Data regarding the long-term outcome of children exposed to maternal cancer with or without treatment during gestation are lacking. Multiple factors, such as maternal illness and nutrition, diagnostic tests, cancer management, and high levels of maternal stress, may negatively influence fetal development. Notably, fetal growth is a multi-aspect process with various stages that may be adversely affected by maternal cancer and its management.

Deciding on the optimal treatment approach towards cancer diagnosed during gestation is frequently a real challenge for physicians. An increased awareness of the feasibility and safety of cancer treatment during pregnancy has enabled more women to receive antenatal treatment and has resulted in more live births and less iatrogenic preterm deliveries [8]. Of note, surgical tumor resection, potentially combined with anthracycline-based chemotherapy administration after the first trimester, is the standard of care for solid tumors, such as early stage pregnancy-associated breast cancer (PABC). However, a multidisciplinary therapeutic approach in specialized centers of expertise is certainly required in order to achieve balance between the maternal benefit and the fetal risk [9,10,11]. Even though neonatal and infant outcomes after oncological treatment during pregnancy seem reassuring, prenatal exposure to anti-cancer drugs remains controversial; further prospective studies on the long-term follow-up until the adulthood of children exposed to maternal chemotherapy should be conducted, as neurocognitive impairment, secondary carcinogenesis and infertility may appear later in life.

This review aims to systematically summarize all existing data from the literature regarding the effect of prenatal exposure to chemotherapy on fetal brain growth and child development. All studies evaluating the neurodevelopment of children in utero exposed to antineoplastic agents will be meticulously analyzed. Solid data on the neurocognitive outcome of children after in utero exposure to chemotherapy may provide both the patients and the physicians with the information necessary for shared decision making when cancer is diagnosed during pregnancy.

## 2. Materials and Methods

All eligible articles included in this review were identified in the Medline/PubMed bibliographical database and the research was conducted according to the PRISMA guidelines [12]; the end-of-search date was 23 October 2020. The search strategy consisted of the following keywords: (maternal OR partum OR gestational OR pregnancy) AND (carcinoma OR cancer OR neoplasm) AND (perinatal OR fetal OR infant OR pediatric) AND (neurodevelopment OR neurodevelopmental OR neurocognitive OR development OR developmental) AND (outcome OR evaluation) AND (in utero OR prenatal) AND exposure AND chemotherapy. Additionally, an investigation of publications indexed in various databases, such as Science Direct, Scopus, and Google Scholar, was conducted, and no further well-defined studies were detected. Furthermore, in order to identify any additional eligible articles, reference lists were also meticulously examined, resulting in a total of 17 articles to be included.

The articles included in this systematic review had to meet certain inclusion criteria: (1) studies highlighting the neurodevelopmental outcome of children after in utero exposure to chemotherapy; (2) studies focusing on the developmental testing of children prenatally exposed to anti-cancer agents based on validated scales or questionnaires (e.g., Bayley Scales of Infant Development, Wechsler Preschool and Primary Scale of Intelligence); (3) articles written in the English language. Publications were excluded if they met one or more of the following criteria: (1) studies not clearly defining the methodology of neurodevelopmental outcome evaluation of children in utero exposed to chemotherapy; (2) animal studies without subsequent validation in human specimens; (3) reviews of literature, Ph.D. summaries, comments, letters or duplicate publications.

In case of overlapping publications emerging from the same study, the larger size study was evaluated, unless additional information was provided in the subsequent manuscripts; in this case, all articles were eligible and were analyzed independently.

From each of the eligible studies, data regarding the maternal cancer as well as the neurodevelopmental outcome of children in utero exposed to chemotherapy were extracted, including the following: first author, year of publication, type of maternal cancer diagnosed during pregnancy, chemotherapy schemes administered during pregnancy, number of pregnant patients treated with anti-cancer agents, median maternal age at diagnosis, trimester of prenatal exposure to chemotherapy, number of livebirths evaluated after in utero exposure to chemotherapy, perinatal outcome (median/mean of gestational age at delivery, preterm births, small for gestational age newborns), median length of follow-up, testing age, neurodevelopmental testing method (e.g., scales, questionnaires), and neurodevelopmental outcome.

## 3. Results

**Figure 1:**
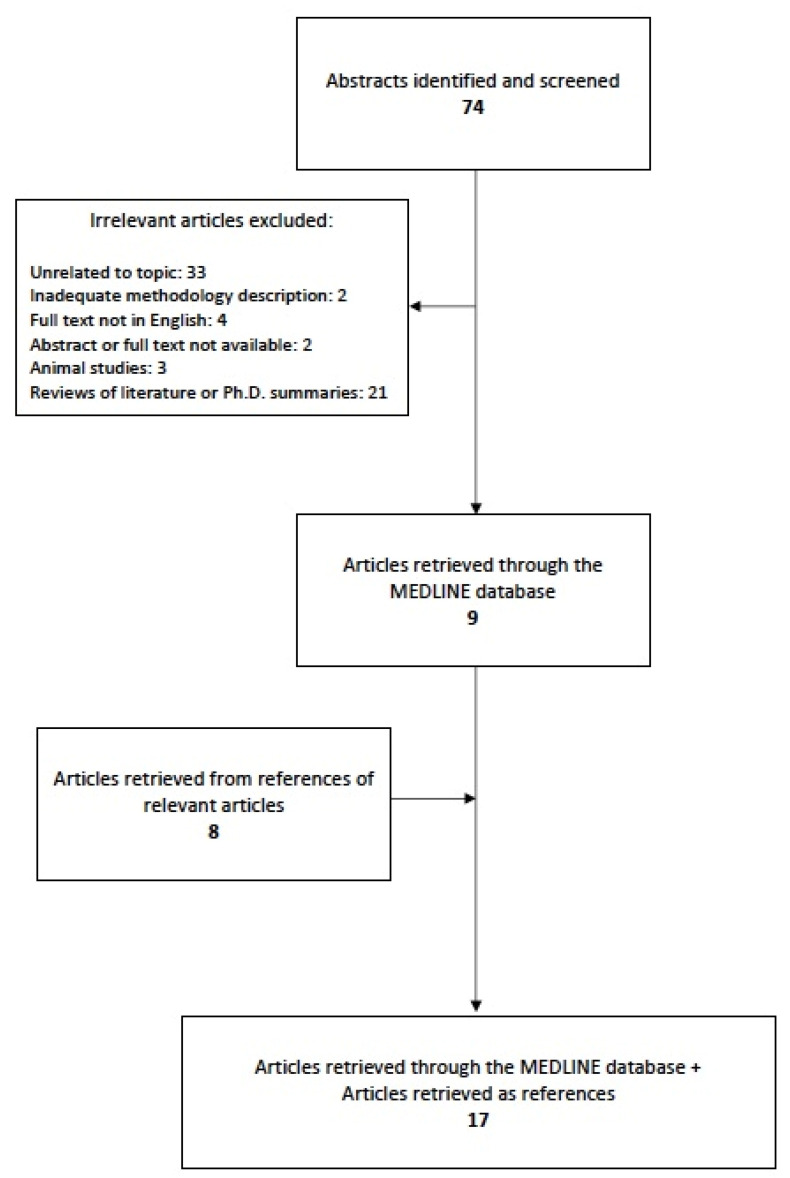
Stages of the search strategy.

The previously described search strategy retrieved in total 74 articles. Of these, 65 were omitted according to the exclusion criteria and nine were considered eligible. While investigating the references of the relevant reviews and eligible studies, eight more articles were added. Overall, 17 articles were entitled eligible for this systematic review, as illustrated in Figure 1. All studies included in our review retrieved retrospective data regarding the maternal disease, whereas the neurocognitive outcome of their children was evaluated in a prospective manner. A summary of the studies describing the type of maternal cancer and the chemotherapy schemes administered during pregnancy while evaluating the neurodevelopmental outcome of children in utero exposed to chemotherapy is demonstrated in Table 1 and Table 2.

Breast cancer and hematological cancer (e.g., acute leukemia, Hodgkin’s lymphoma, non-Hodgkin’s lymphoma) were the most frequently diagnosed malignancies during gestation [13,14,15,16,17,18,19,20,21,22,23,25,26,27,28,29]. Additionally, several cases of cervical, ovarian, brain and gastric cancer were also reported [13,14,20,21,22,24,26,27,28,29]. Some studies focused on a single type of pregnancy-related cancer analyzing its treatment along with the maternal and the fetal outcomes [15,16,17,18,23,24,25], whereas other studies integrated data regarding multiple cancer types, as shown in Table 1 [13,14,20,21,22,26,27,28,29]. Furthermore, various chemotherapy regimens were administered during the course of pregnancy, depending on the type of maternal cancer diagnosed; a combination of anthracycline-based regimens was most commonly used [13,14,15,16,17,18,19,20,21,22,23,24,25,26,27,28]. Cyclophosphamide, 5-fluorouracil, taxanes, and platinum derivatives were also regularly administered [13,14,15,16,17,18,19,20,21,22,23,24,25,26,27,28]. In the majority of the studies included in the review, the antineoplastic agents were administered in the second and third trimester of pregnancy [13,14,20,21,22,23,24,25,26,27,28,29]; however, in all studies mainly investigating hematological malignancies, chemotherapy was administered in the first trimester as well [15,16,17,18,19].

As illustrated in Table 2, the long-term neurodevelopmental outcome was evaluated only in livebirths prenatally exposed to chemotherapy. Each study was based on a combination of different developmental testing methods; validated neurodevelopmental scales (e.g., Bayley Scales of Infant Development, Wechsler Preschool and Primary Scale of Intelligence), various behavioral inquiries (e.g., Child Behavior Checklist, Behavior Rating Inventory of Executive Function), well-defined general health and education questionnaires filled by the parents or the caregivers, school performance questionnaires filled by the teachers, brain MRI neuroimaging, and/or electroencephalography (EEG) combined with event-related potentials (ERP).

Several studies applied the Bayley Scales of Infant Development in children aged 18 and/or 36 months in order to evaluate the neurocognitive outcomes after in utero exposure to cytotoxic chemotherapy [13,14,22,24,26]. No major cognitive abnormalities were observed in children prenatally exposed to anti-cancer agents. Of note, a few years ago, Amant et al. demonstrated the negative prognostic effect of prematurity on the cognitive skills of the 1.5–3 years of age cohort [13,14], whereas Cardonick et al. and the most recent studies by Amant et al., examining children at the age of 6 years, did not confirm such correlation [22,27,28]. Moreover, the type of chemotherapy or the number of chemotherapy cycles administered during pregnancy had no impact on the neurodevelopment of children in utero exposed to antineoplastic agents according to a study by Amant et al. [14].

Depending on the testing age, Wechsler Intelligence Scales were used in multiple versions; Wechsler Preschool and Primary Scale of Intelligence, Wechsler Intelligence Scale for Children, Wechsler Adult Intelligence Scale, Wechsler Individual Achievement Test [13,15,16,20,21,22,24,27,28,29]. Interestingly, Amant et al. reported a disharmonic IQ profile in chemotherapy-exposed children older than 6 years of age, while identifying a significant difference between verbal and performance IQ scores [13]. Additionally, the studies by both Vandenbroucke et al. and Van Gerwen et al. also highlighted significantly lower verbal IQ score in children exposed to maternal chemotherapy, especially in those whose mothers died after delivery [27,28,29]. Nevertheless, the majority of the studies proved that intelligence test results were within normal ranges and in accordance with children’s social and economic background [15,16,20,21,22,24]. As stated by Vandenbroucke et al., full scale IQ was not related either with the gestational age of exposure in the second or third trimester, or with the number of chemotherapy cycles administered during pregnancy [27,28].

In order to evaluate the impact of maternal chemotherapy on child behavior and executive function, the Child Behavior Checklist (CBCL) and/or the Behavior Rating Inventory of Executive Function (BRIEF) were completed by the parents or the caregivers [13,21,22,24,27,28,29]. In one of the most recent studies, Van Gerwen et al. demonstrated that children prenatally exposed to anti-cancer drugs were characterized by significantly weaker emotion control skills when compared to non-exposed children of the same age [29]. Additionally, internalizing, externalizing, and total behavior problems were reported in six out of twenty-one CBCLs filled out in a study by Amant et al., while no correlation with prematurity was proven [13]. Similarly, Cardonick et al. identified significantly higher rates of internalizing problems in older children previously exposed to maternal chemotherapy when compared to the age-matched unexposed control group included in the study [22]. 

Aiming to define the long-term neurodevelopmental outcome after prenatal exposure to antineoplastic agents, certain studies were based solely on general health and education questionnaires or school performance questionnaires, whereas other studies included those surveys as part of the overall evaluation [13,14,15,16,17,18,19,23,25,27,28,29]. No crucial abnormalities were observed in conduct or educational performance through the abovementioned questionnaires. Only a few cases were reported by Hahn et al. and Murthy et al., two studies emerging from the same cohort; some children required special attention in school and others were characterized by developmental milestone delays [23,25].

Complementary to the neurodevelopmental scales, three particular studies included in the review focused on brain MRI neuroimaging and ERP/EEG testing in order to investigate potentially damaged brain areas and correlate these findings with neurocognitive impairment [20,21,26]. According to Passera et al., no statistically significant differences between children exposed to maternal chemotherapy and controls were observed in both the total and the regional brain volumes and no correlation was found between the brain volumes and the neurodevelopmental outcome [26]. Even though Blommaert et al. demonstrated that prenatal exposure to chemotherapy had a negative impact on response inhibition and spatial attention, differences in brain MRI neuroimaging were not related to the neurocognitive outcome as well [20,21].

Further data extracted from all eligible studies regarding the long-term neurodevelopmental outcome of children prenatally exposed to chemotherapy are presented in Table 1 and Table 2. A critically assessed evaluation of the eligible studies is shortly presented in the discussion section.

## 4. Discussion

The long-term neurodevelopmental effect of in utero exposure to anti-cancer agents, which is an important aspect of chemotherapy treatment safety, has only recently been addressed. We conducted a comprehensive systematic literature review that resulted in the identification of 17 studies exploring the impact of prenatal exposure to chemotherapy on the neurodevelopment of children. Our results demonstrate that no major consequences on long-term neurodevelopmental outcome of children after in utero exposure to chemotherapy have been detected; specific facets of each individual study should be further discussed.

In the past, fear of cytotoxic chemotherapeutic impact on the developing fetus has prevented clinicians from starting oncological management during gestation and resulted in pregnancy termination, medically-induced preterm delivery or delay of maternal treatment [30]. Current guidelines, however, taking into consideration the favorable safety profile of specific chemotherapeutic agents (e.g., anthracyclines, taxanes), recommend systemic chemotherapy administration during the second and third trimester of pregnancy with close monitoring of both the mother and the fetus [31]. Accordingly, anthracycline-based regimens combined with taxanes or platinum derivatives were most commonly administered in the studies included in our review [13,14,15,16,17,18,19,20,21,22,23,24,25,26,27,28], resulting in longer gestations, more livebirths and less treatment delays.

The placenta and the fetal blood–brain barrier constitute the primary defense mechanism of the fetus against toxic chemotherapeutic agents. Passive diffusion represents the main transfer mechanism of the placenta; molecule penetration follows its concentration gradient and is determined by its physiochemical characteristics such as lipid solubility, polarity and molecular weight. Additionally, active transport by protein pumps (e.g., P-glycoprotein) works against the concentration gradient in the maternal and the fetal blood flow and provides an energy-requiring link between the two circulations [32]. Of note, the effect of the placental protection differs by chemotherapeutic agent, with a high penetration of platinum-based therapies (57% for carboplatin), but a low passage for taxanes (1% for paclitaxel and not detectable for docetaxel) and anthracyclines (4% for epirubicin and 8% for doxorubicin) [30]. Our review also highlights the safety profile of anthracycline-based regimens, as no congenital abnormalities were reported and no major neurocognitive impairment was observed. Furthermore, tight junctions, low rates of transcytosis, and expression of specialized influx and efflux transporters on the fetal blood–brain barrier are present early in the embryological development [33]. However, several possible underlying mechanisms of neurotoxicity have also been reported; immature fetal metabolism, oxidative stress, inflammation, and anti-angiogenic effect [34].

Additionally to the preferable chemotherapy scheme, the timing of drug administration during the course of pregnancy and embryogenesis is crucial. When cytotoxic drugs are administered in the first ten days after fertilization, the result is an all-or-nothing phenomenon depending on the number of disrupted cells. When cytotoxic drugs are administered during the phase of organogenesis that starts at day 10 and is completed at week 8, there is high risk of teratogenic effects and increased frequency of major congenital malformations. Hence, chemotherapy is contraindicated during the first trimester of pregnancy [35]. In our review, fetuses were mainly exposed to maternal chemotherapy during the second and third trimester of pregnancy, with the exception of a few studies evaluating hematological malignancies and child development before the last decade [15,16,17,18,19]. Notably, despite the early prenatal exposure to anti-cancer agents, the incidence of congenital malformations in these cases was similar to the general population and no significant abnormalities in long-term cognitive testing were reported.

Interestingly, central nervous system (CNS) development starting at week 5 remains vulnerable throughout the whole pregnancy, while it continues even during the postnatal period. Thus, potential harmful effect of cytotoxic drugs on fetal brain growth may occur later in pregnancy and result in neurocognitive impairment and poor behavioral or academic performance, known as the “chemo-brain” effect [30]. Similarly to adults and children cancer survivors who received chemotherapy, of major concern is the potential toxicity on the frontal lobe that is responsible for emotions and executive functions such as attention control and working memory [36].

Our review demonstrates that no significant impairment in neurocognitive development of children prenatally exposed to anti-cancer drugs has been detected. However, various abnormalities were observed in child behavior and executive function after prenatal exposure to chemotherapy during the second and third trimester; weak emotional regulation, and high rates of internalizing, externalizing, and total behavior problems were observed. These findings may also be attributed to antenatal maternal stress, as several studies have demonstrated that maternal psychosocial functioning during pregnancy is highly associated with child cognition [37,38,39]. Thus, it is possible that child behavior and executive function are both influenced by the stress caused by cancer diagnosis during pregnancy. In addition, the negative effect of prematurity on cognitive development mentioned in the studies by Amant et al. included in our review is well-established in the literature [40,41]. Therefore, iatrogenic prematurity should be avoided when possible and cancer treatment, including chemotherapy, should be offered during pregnancy in order to prevent systemic spread and preserve the long-term neurodevelopmental outcome at the same time.

Among the limitations of this review, it should be stressed that our conclusions are based on studies evaluating child neurodevelopment at different testing ages using various assessment tools (e.g., neurocognitive scales, questionnaires), studies examining children exposed to different types of maternal cancer and chemotherapy treatment plans, and studies based on small cohorts, some of which do not include control groups or have short follow-up periods. Furthermore, the number of eligible studies was limited due to the rarity of pregnancy-related cancer; thus, further research is needed in order to confirm the abovementioned findings.

## 5. Conclusions

In conclusion, further research in the field of prenatal exposure to maternal cancer is highly recommended in order to obtain sufficient evidence and formulate pregnancy-related cancer management guidelines. Even though current guidelines suggest close monitoring of the fetal and the neonatal development, a clearly defined approach to the long-term follow up after prenatal exposure to anti-cancer drugs is not available [31]. The data originating from the 17 studies included in our review suggest that chemotherapy administration during the second and third trimester of pregnancy is feasible with no major consequences on the neurodevelopmental fetal outcome; nevertheless, longer and more thorough follow-up is certainly required in order to draw firm conclusions. Due to the rarity of the disease, large-scale multicenter prospective studies with longer follow-up until adulthood will provide valuable insight into the long-term neurodevelopmental outcome after in utero exposure to anti-cancer drugs. Currently, efforts toward this direction are mainly represented by the International Network on Cancer, Infertility, and Pregnancy (INCIP), which is one of the largest international registries promoting research on pregnancy-related cancer and collecting oncological, obstetrical, and perinatal data. Last but not least, research focusing on a single cancer type and specific chemotherapeutic agents administered during pregnancy will elucidate their distinct impact on fetal brain growth. Confounding factors such as maternal stress and nutrition should also be evaluated as the question of whether the maternal psychosocial status or the cancer management plan has a more significant impact on child neurodevelopment remains to be answered. Viewed collectively, the abovementioned evidence will also enable clinicians to confidently advice the parents on how to accurately evaluate the neurocognitive outcome of their child after in utero exposure to anti-cancer drugs through the monitoring of his/her general health, behavior, and school performance.

## Figures and Tables

**Table 1:** Summary of the studies describing the type of maternal cancer and the chemotherapy scheme(s) administered during pregnancy.

Author	Type of Maternal Cancer Diagnosed during Pregnancy	Chemotherapy Scheme(s) Administered during Pregnancy	Median Maternal Age (y) at Diagnosis	Trimester of Prenatal Exposure to Chemotherapy
Amant et al., 2012 [13]	Breast cancer (35) Hematological malignancy (18) Ovarian cancer (6) Cervical cancer (4) Basal cell cancer (1) Brain tumor (1) Ewing sarcoma (1) Colorectal cancer (1)Nasopharyngeal cancer (1)	(F)AC or (F)E(C) (34/68), MOPP or ABV (2/68), ABVD (5/68), CHOP-Rituximab (4/68), Cisplatin +/− [5-FU or Cyclophosphamide] (8/68), Paclitaxel + Cis/Carboplatin (3/68), Paclitaxel or Docetaxel (2/68), CMF (2/68), ALL Hovon scheme (5/68), Idarubicin + Ara C (2/68), Daunorubicin + Ara C (1/68), Temozolomide (1/68), 5-FU (1/68), Vincristine + Doxorubicin + Methotrexate (1/68), Amsacrine + Tenoposide (1/68), VIM (without methotrexate) (1/68)	32.9	2nd and 3rd trimester
Amant et al., 2015 [14]	Breast cancer (69)Hematological malignancy (20) Cervical cancer (10) Ovarian cancer (9) Brain cancer (3) Colon cancer (3) Gastric cancer (2) Renal cancer (1) Tongue cancer (2) Lung cancer (1) Thyroid cancer (2) Melanoma (1) Ewing sarcoma (1) Soft tissue sarcoma (1)	(F)AC or (F)E(C) (58/93), ABVD (7/93), CHOP-Rituximab (7/93), Cisplatin +/− Epirubicin (6/93), Carboplatin +/− 5-FU (1/93), Paclitaxel + Cis/Carboplatin (9/93), Paclitaxel or Docetaxel (14/93), Hovon 37 (1/93), Temozolomide (1/93), Idarubicin + Ara C (1/93), Daunorubicin + Ara C (1/93), 5-FU (1/93), VIM (without methotrexate) (1/93)	33.4	2nd and 3rd trimester
Avilés et al., 1988 [15]	Acute leukemia (23)	Combination of: Triamcinolone, 6-Mercaptopurine, Cyclophosphamide, Methotrexate, Vincristine, Prednisone, Ara C, Etoposide, Doxorubicin	N/A	1st, 2nd and 3rd trimester
Avilés et al., 1991 [16]	Acute leukemia (7) Non-Hodgkin’s lymphoma (18) Hodgkin’s lymphoma (14) Chronic granulocytic leukemia (4)	Combination of: Vincristine, Prednisone, Doxorubicin, 6-Mercaptopurine, Methotrexate, Cyclophosphamide, Ara C CHO-Bleomycin (2/18), CHOP +/− [Bleomycin +/− Ara C +/− Methotrexate] (12/18), CEOP-Bleomycin +/− [Ara C +/− Methotrexate] (4/18) MOPP (4/14), ABVD (5/14), ABVD + MOPP (3/14), ABVD + PDN (2/14) Combination of: Busulfan, Prednisone, 6-Mercaptopurine	24.0 29.0 28.0 30.0	1st, 2nd and 3rd trimester
Avilés et al., 2001 [17]	Acute leukemia (29) Malignant lymphoma (29) Hodgkin’s lymphoma (26)	COPA (10/29), Combination of: Ara C + [Daunorubicin (4/29) or Mitoxantrone (3/29) or Doxorubicin (8/29) or Idarubicin (4/29)] CHOP-Bleomycin (29/29) MOPP (10/26), ABVD (10/26), EBVD (4/26), MOPP + [ABVD or ABD] (2/26)	29.6 N/A N/A	1st, 2nd and 3rd trimester
Avilés et al., 2012 [18]	Acute leukemia (14) Non-Hodgkin’s lymphoma (25) Hodgkin’s lymphoma (19)	COPA (6/14), Ara C + Anthracycline (8/14) CHOP (17/25), CHOP-Rituximab (3/25), Intensive (5/25) ABVD (12/19), MOPP (5/19), MOPP + ABVD (2/19)	26.8 29.3 22.0	1st trimester
Blatt et al., 1980 [19]	Acute leukemia (2) Hodgkin’s lymphoma (1) Undifferentiated sarcoma (1)	Ara C (1/2), Prednisone + Vincristine + Methotrexate + 6-Mercaptopurine (1/2) MOPP (1/1) Cyclophosphamide + Adriamycin + Vincristine + AMSA (1/1)	N/A	1st, 2nd and 3rd trimester
Blommaert et al., 2019 [20]	Breast cancer (12) Cervical cancer (2) Hodgkin’s lymphoma (2) Non-Hodgkin’s lymphoma (1) Acute leukemia (2) Colon carcinoma (1)	FEC or FAC (7/20), Cyclophosphamide + Doxorubicin (3/20), ABVD (2/20), Platinum derivative (2/20), 5-FU (1/20), Ara C + Vincristine + Mitoxantrone (1/20), Cyclophosphamide + Methotrexate (1/20), Daunorubicin + Ara C (1/20), CHOP-Rituximab (1/20), Trastuzumab (1/20)	34.0 (at birth)	2nd and 3rd trimester
Blommaert et al., 2020 [21]	Breast cancer (25) Cervical cancer (3) Ovarian cancer (1) Hodgkin’s lymphoma (3) Tongue cancer (3) Leukemia (2) Brain tumor (2) Melanoma (1) Kidney carcinoma (1) Colon cancer (1)	FEC or FAC (11/30), Cyclophosphamide + [Doxorubicin or Epirubicin] (7/30), ABVD (3/30), Cisplatin (3/30), Carboplatin + 5-FU (2/30), 5-FU (1/30), Daunorubicin + Ara C (1/30), Epirubicin (1/30), Temozolomide (1/30)	32.0 (at birth)	2nd and 3rd trimester
Cardonick et al., 2015 [22]	Breast cancer (26) Ovarian cancer (4) Hodgkin’s lymphoma (4) Acute leukemia (1)	Doxorubicin + Cyclophosphamide (22/26) +/− [5-FU (3/26) or Paclitaxel (1/26)] Cisplatin + Paclitaxel (2/4), Etoposide + Cisplatin + Bleomycin (1/4), Carboplatin + Paclitaxel (1/4) Doxorubicin + Bleomycin + Vinblastine + Dacarbazine (4/4) Cyclophosphamide + Daunorubicin + Vincristine + L-asparaginase + Cytarabine + 6-Mercaptopurine + Intrathecal Methotrexate (1/1)	N/A	2nd and 3rd trimester
Hahn et al., 2006 [23]	Breast cancer (57)	Combination of: Cyclophosphamide (36/57), Doxorubicin (36/57), 5-FU (35/57)	33.5 (mean)	2nd and 3rd trimester
Maggen et al., 2020 [24]	Gastric cancer (13)	5-FU or FOLFOX or [Carboplatin + Paclitaxel] (13/13)	31.7	2nd and 3rd trimester
Murthy et al., 2014 [25]	Breast cancer (81)	FAC (81/81)	N/A	2nd and 3rd trimester
Passera et al., 2019 [26]	Breast cancer (24) Ovarian cancer (1) Cervical cancer (1) Lung cancer (1) Nasopharyngeal cancer (1) Hodgkin’s lymphoma (2) Non-Hodgkin’s lymphoma (1)	[Anthracyclines (Epirubicin 26/31) + Cyclophosphamide] (31/31)	35.0 (mean)	2nd and 3rd trimester
Vandenbroucke et al., 2020 [27] and Van Gerwen et al., 2020 [28]	Breast cancer (69) Hematological malignancy (20) Cervical cancer (10) Ovarian cancer (10) Brain cancer (4) Oral cavity and oropharyngeal cavity cancer (4) Nasopharyngeal cancer (1) Gastric cancer (2) Colon cancer (1) Melanoma (2) Thyroid cancer (1) Soft tissue sarcoma (1) Kidney carcinoma (1) Lung cancer (1)	(F)AC or (F)E(C) (58/93), ABVD (5/93), CHOP-Rituximab (5/93), Cisplatin +/− Epirubicin (9/93), Carboplatin +/− 5-FU or Cisplatin +/− 5-FU (3/93), Paclitaxel + Cis/Carboplatin (7/93), Paclitaxel or Docetaxel (12/93), Hovon 30 or 70 or 42A (2/93), Temozolomide (1/93), Idarubicin + Ara C (2/93), 5-FU (1/93), CMF (1/93)	N/A	2nd and 3rd trimester
Van Gerwen et al., 2020 [29]	Breast cancer (26) Cervical cancer (3) Tongue cancer (2) Gastric cancer (1) Hodgkin’s lymphoma (1) Non-Hodgkin’s lymphoma (1)	N/A	N/A	2nd and 3rd trimester

Abbreviations: FAC/FEC: 5-fluorouracil, adriamycin/epirubicin, cyclophosphamide; ABV: adriamycin, bleomycin, vinblastine; ABVD: adriamycin, bleomycin, vinblastine, dacarbazine; CHOP: cyclophosphamide, adriamycin, vincristine, prednisone; 5-FU: 5-fluorouracil; Ara C: cytosine arabinoside; VIM: ifosfamide, etoposide, methotrexate; CHO: cyclophosphamide, adriamycin, vincristine; CEOP: cyclophosphamide, epidoxorubicin, vincristine, prednisone; MOPP: mechloretamine, vincristine, prednisone, procarbazine; PDN: prednisone; COPA: cyclophosphamide, vincristine, prednisone, adriamycin; EBVD: epirubicin, bleomycin, vinblastine, dacarbazine; ABD: adriamycin, bleomycin, dacarbazine; AMSA: 4′-(9-acridinyloamino)methanesulphon-m-anisidiate) FOLFOX: folinic acid, fluorouracil, oxaliplatin; CMF: cyclophosphamide, methotrexate, 5-fluorouracil; N/A: non-available.

**Table 2:** Summary of the studies describing the neurodevelopmental outcome of children after in utero exposure to chemotherapy.

Author	Children Evaluated	Perinatal Outcome	Median f/u	Testing Age	Neurodevelopmental Testing Method	Neurodevelopmental Outcome
Amant et al., 2012 [13]	70	Median GA (w): 35.7 Preterm: 47/70 SGA: 14/70	22.3 months	Birth 18 months 5–6, 8–9, 11–12, 14–15, or 18 years	Clinical neurological examination General health and education questionnaire Bayley Scales of Infant Development Wechsler Preschool and Primary Scale of Intelligence Snijders–Oomen Nonverbal Intelligence Test Children’s Memory Scale Child Behavior Checklist Wechsler Intelligence Scale for Children Test of Everyday Attention for Children Auditory Verbal Learning Test Wechsler Adult Intelligence Scale	Neurocognitive outcome within normal range. Negative prognostic effect of prematurity on cognitive development (Bayley or IQ score). Severe neurodevelopmental delay in two children, both members of a twin pregnancy. Although a clinical picture suggested a syndromal entity, an effect of chemotherapy cannot be excluded. Significant difference between verbal and performance IQ score in children older than 6 years of age (Wechsler Intelligence Test). Internalizing, externalizing, and total behavior problems reported in 6/21 children (CBCL). No significant correlation with prematurity.
Amant et al., 2015 [14]	129 (31 of whom included in previously published results [13]) 96 of whom in utero exposed to chemotherapy	Median GA (w): 36 Preterm: 79/129 ** SGA: 28/127 ** SGA: 24/95	21 months	18 months and/or 36 months	Clinical neurological examination General health and education questionnaire Bayley Scales of Infant Development	Negative prognostic effect of prematurity on cognitive development. Cognitive outcomes not significantly different among the prenatal-exposure group and the control group. No differences according to the type of chemotherapy or the number of chemotherapy cycles administered.
Avilés et al., 1988 [15]	17	Median GA (w): N/A Preterm: N/A SGA: N/A	6 years	4–22 years	Clinical neurological examination School performance questionnaire filled by teachers Wechsler Intelligence Scale for Children Bender–Gestalt Test for Young Children	No abnormalities in conduct or school performance. No differences in cognitive testing.
Avilés et al., 1991 [16]	43	Median GA (w): 38 Preterm: 8/43 SGA: N/A	N/A	3–19 years	Clinical neurological examination School performance questionnaire filled by teachers Wechsler Intelligence Scale for Children Bender–Gestalt Test for Young Children	No abnormalities in conduct or school performance. Weschler Intelligence Test results within normal ranges. Development showed no difference in children of the same social and economic background.
Avilés et al., 2001 [17]	84	Median GA (w): N/A Preterm: N/A SGA: 0/84	18.7 years	6–29 years	Clinical neurological examination School performance questionnaire filled by teachers	Neurological examination, psychological evaluation, educational performance and behavior of children exposed to maternal chemotherapy considered normal.
Avilés et al., 2012 [18]	54	Median GA (w): N/A Early preterm: 4/54 SGA: 10/54	22.4 years	Birth 3, 6, 12, 18, 24 months 3, 5, 7, 10, 15, 20 years	Clinical neurological examination School performance questionnaire filled by teachers	Intelligence test, including verbal and performance IQ score, within normal ranges. Academic development according to age, economic and social status.
Blatt et al., 1980 [19]	3	Median GA (w): N/A Preterm: 0/3 SGA: 0/3	7 years	2.5–8 years	Clinical neurological examination Denver Developmental Screening Test School performance questionnaire filled by teachers	Growth, development, and school performance were normal. No major abnormalities.
Blommaert et al., 2019 [20]	20	Median GA (w): 35.6 Preterm: N/A SGA: N/A	9.18 years	9 years	Wechsler Intelligence Scale for Children Event-related potentials (ERP) Electroencephalography (EEG)	Prenatal exposure to chemotherapy had a negative impact on response inhibition and spatial attention. Prenatal exposure to chemotherapy and prematurity might both alter the development of conflict monitoring.
Blommaert et al., 2020 [21]	42 30 of whom in utero exposed to chemotherapy	Median GA (w): 36.3 ** Preterm: 26/42 ** SGA: 7/42 **	9.19 years **	9 years	Wechsler Intelligence Scale for Children Child Behavior Checklist Behavior Rating Inventory of Executive Function Brain MRI	All psycho-behavioral measures within normal ranges, though children born after cancer-complicated pregnancies showed a slightly lower total IQ score. Psycho-behavioral parameters not significantly related to any of the brain differences in MRI neuroimaging. Differences in brain MRI neuroimaging observed within chemotherapy subgroup when exposed to platinum derivatives or anthracyclines. No significant correlation with neurocognitive outcome.
Cardonick et al., 2015 [22]	35	Mean GA (w): 36.7 Preterm: 51.4% SGA: 1/35	Mean value: 4.5 years	18 months-10.4 years	Bayley Scales of Infant Development Wechsler Preschool and Primary Scale of Intelligence Wechsler Intelligence Scale for Children Wechsler Individual Achievement Test Child Behavior Checklist	No significant differences in cognitive skills, academic achievement, or behavioral competence between the chemotherapy-exposed group and the unexposed children. Premature birth more prevalent in the chemotherapy-exposed group. No correlation with developmental outcome. Older children demonstrated significantly higher rates of internalizing behavior problems.
Hahn et al., 2006 [23]	52	Mean GA (w): 37 Preterm: N/A SGA: N/A	38.5 months	2-157 months	General health and education questionnaire	Of the school-age children (*n* = 18), only two required special attention in school: one child had attention deficit disorder, whereas the other had Down-syndrome.
Maggen et al., 2020 [24]	10 6 of whom in utero exposed to chemotherapy	Median GA (w): 32 ** Preterm: 4/6 SGA: 2/6	N/A	4, 6, 15, 18 months, 3, 6 years	Clinical neurological examination Bayley Scales of Infant and Toddler Development Child Behavior Checklist Behavior Rating Inventory of Executive Function Wechsler Preschool and Primary Scale of Intelligence Children’s Memory Scale Amsterdam Neuropsychological Tasks	No neurocognitive abnormalities.
Murthy et al., 2014 [25]	81 (Update on previously published initial report in 2006 [23])	Mean GA (w): 37 Preterm: 28/81 SGA: N/A	7 years	<1–22 years	General health and education questionnaire	6/50 survey responders: children with developmental milestone delays (3/50 childhood language delays). No significant cognitive abnormalities. 37 children enrolled in pre-school through college: 3/37 reading delays, 4/37 difficulties in school, 1/37 difficulty with attention span.
Passera et al., 2019 [26]	31 (10 of whom included in previously published results [14])	Mean GA (w): 36.3 Preterm: 15/31 SGA: 5/31	Mean value: 41.1 weeks (at brain MRI) Mean value: 19.8 months (at Bayley Scales)	Mean post-menstrual age: 41.1 weeks 18 months	Clinical neurological examination Brain MRI Bayley Scales of Infant Development	No statistically significant differences between children exposed to chemotherapy and controls in both the total and the regional brain volumes (brain MRI). Exposed children with normal Bayley scores. No significant correlation between the brain volumes and the neurodevelopmental outcome. No correlation between the neurodevelopmental outcome and the cumulative dosage of epirubicin administered.
Vandenbroucke et al., 2020 [27] and Van Gerwen et al., 2020 [28]	132 (12 of whom included in previously published results [13]) 97 of whom in utero exposed to chemotherapy	Median GA (w): 36.1 ** Preterm: 80/132 ** SGA: 14/97	6.1 years	6 years	Clinical neurological examination General health and education questionnaire Wechsler Preschool and Primary Scale of Intelligence Wechsler Intelligence Scale for Children Snijders–Oomen Nonverbal Intelligence Test Children’s Memory Scale Amsterdam Neuropsychological Tasks Child Behavior Checklist	Although within normal range, statistically significant differences in mean verbal IQ and visuospatial long-term memory; lower scores in children prenatally exposed to maternal chemotherapy. Verbal IQ more affected in children whose mothers died than in children with surviving mothers. No correlation of prematurity with cognitive outcome. Full scale IQ not related to GA in the chemotherapy-exposed group or to the number of chemotherapy cycles administered during pregnancy.
Van Gerwen et al., 2020 [29]	37	Median GA (w): 35.6 Preterm: N/A SGA: N/A	6.1 years	6 years	Behavior Rating Inventory of Executive Function General health and education questionnaire Wechsler Preschool and Primary Scale of Intelligence	All outcome scales within normal ranges (BRIEF). Significant between-group difference in emotional control; weaker emotion regulation skills in children prenatally exposed to chemotherapy. Significantly lower verbal IQ score in children prenatally exposed to chemotherapy.

Abbreviations: GA: gestational age; SGA: small for gestational age; ERP: event-related potentials; EEG: electroencephalography; CBCL: Child Behavior Checklist; BRIEF: Behavior Rating Inventory of Executive Function; MRI: magnetic resonance imaging, N/A: non-available.** Children exposed in general to maternal cancer management (i.e., chemotherapy and/or surgery and/or radiotherapy).

## Abbreviations

PABC: pregnancy-associated breast cancer; NIPT: non-invasive prenatal testing; MRI: magnetic resonance imaging; EEG: electroencephalography; ERP: event-related potentials; IQ: intelligence quotient; CBCL: Child Behavior Checklist; BRIEF: Behavior Rating Inventory of Executive Function; CNS: central nervous system; INCIP: International Network on Cancer, Infertility, and Pregnancy.
